# MicroRNA-mediated post-transcriptional regulation of *Pinus pinaster* response and resistance to pinewood nematode

**DOI:** 10.1038/s41598-022-09163-3

**Published:** 2022-03-25

**Authors:** Inês Modesto, Vera Inácio, Yves Van de Peer, Célia M. Miguel

**Affiliations:** 1 ITQB NOVA, Universidade Nova de Lisboa, Oeiras, Portugal; 2 iBET, Oeiras, Portugal; 3 Department of Plant Biotechnology and Bioinformatics, Ghent University, Ghent, Belgium; 4 Faculdade de Ciências, Biosystems & Integrative Sciences Institute, Universidade de Lisboa, Lisbon, Portugal; 5 VIB-UGent Center for Plant Systems Biology, Ghent, Belgium; 6 Department of Biochemistry, Genetics and Microbiology, University of Pretoria, Pretoria, South Africa; 7 College of Horticulture, Academy for Advanced Interdisciplinary Studies, Nanjing Agricultural University, Nanjing, 210095 China

**Keywords:** Plant immunity, Plant molecular biology, Plant stress responses, Non-coding RNAs

## Abstract

Pine wilt disease (PWD), caused by the parasitic nematode *Bursaphelenchus xylophilus*, or pinewood nematode (PWN), is a serious threat to pine forests in Europe. *Pinus pinaster* is highly susceptible to the disease and it is currently the most affected European pine species. In this work, we investigated the role of small RNAs (sRNAs) in regulating *P. pinaster*–PWN interaction in an early stage of infection. After performing an artificial PWN inoculation assay, we have identified 105 plant microRNAs (miRNAs) responsive to PWN. Based on their predicted targets, part of these miRNAs was associated with roles in jasmonate-response pathway, ROS detoxification, and terpenoid biosynthesis. Furthermore, by comparing resistant and susceptible plants, eight miRNAs with putative functions in plant defence and resistance to PWN have been identified. Finally, we explored the possibility of bidirectional trans-kingdom RNA silencing, identifying several *P. pinaster* genes putatively targeted by PWN miRNAs, which was supported by degradome analysis. Targets for *P. pinaster* miRNAs were also predicted in PWN, suggesting a role for trans-kingdom miRNA transfer and gene silencing both in PWN parasitism as in *P. pinaster* resistance to PWD. Our results provide new insights into previously unexplored roles of sRNA post-transcriptional regulation in *P. pinaster* response and resistance to PWN.

## Introduction

Pinewood nematode (PWN), or *Bushaphelenchus xylophilus*, is a migratory plant-parasitic nematode that causes pine wilt disease (PWD) in several conifer species. PWN is transmitted to healthy trees through the insect vector *Monochamus* spp. while it feeds on the tree’s bark^1,2^. This nematode infects the tree stem, migrating through resin canals and feeding on plant tissues. The progressive destruction of stem tissues leads to the disruption of water flow, causing the wilting and death of the tree.

PWD has become an increasing threat to worldwide conifer forests, especially in Asia and South-eastern Europe, causing economic losses in the forestry industry and having a severe environmental impact^3^. In Europe, PWD was first detected in Portugal in 1999^4^ and has since spread to Spain, despite the sanitary measures implemented^5^. *Pinus pinaster* is the mainly affected species in this region^1,3^.

As a strategy to help mitigate the spreading and damage of PWD, resistant varieties of susceptible *Pinus* species have been developed^6,7^. Breeding programs were successfully implemented for *Pinus thunbergii*, *Pinus densiflora*, and *Pinus massoniana*^6,7^. For *P. pinaster*, the first steps were given in order to select individuals with increased PWN resistance^8,9^.

Plant defence response initiates after the recognition of the pathogen by cell membrane receptor-like kinases (RLKs) or receptor-like proteins (RLPs), activating the pattern-triggered immunity (PTI)^10^. Pathogens and pests can, however, produce effectors that suppress PTI. In turn, plants may recognize these effectors through nucleotide-binding/leucine-rich-repeat (NLR) receptors, initiating the more robust effector-triggered immunity (ETI)^10^. The activation of PTI and ETI trigger immune responses controlled by plant hormones, such as salicylic acid (SA), jasmonic acid (JA), ethylene (ET) or abscisic acid (ABA)^10,11^. In response to PWN inoculation, a transcriptional reprogramming was observed in *P. pinaster* stem tissues^12,13^. This included the differential expression of RLK/RLP and NLR encoding genes, as well as genes involved in secondary metabolism, oxidative stress response, lignin synthesis, and phytohormones signalling pathways. An increase in JA levels was observed after inoculation, while high SA levels were associated with susceptibility. Furthermore, resistant plants showed higher lignification around the inoculation zone when compared to susceptible plants^13^.

Several studies have shown important roles for small non-coding RNAs (sRNAs) in the interaction of host plants with viruses, bacteria, fungi, nematodes, and herbivore insects^14–16^. MiRNAs have been implicated in the regulation of plant hormone synthesis and signalling, callose deposition, expression of *NLR receptors*, and production of secondary metabolites. On the other hand, pathogens’ and pests’ effectors may suppress the plant immune response by reducing the accumulation of sRNAs or interfering with the RNA silencing machinery^14–16^. Furthermore, trans-kingdom RNA silencing has been reported, in which sRNAs encoded by pathogens directly suppress host genes with roles in plant immunity^17–19^. Likewise, transgenic plants expressing exogenous sRNAs/dsRNAs can induce the silencing of genes in pathogens or pests, in a process called host-induced gene silencing (HIGS)^14,15^. Recent studies suggest that naturally occurring plant miRNAs may also be transferred to pathogens and target their genes in order to fight infection^19,20^.

The role of miRNAs in the regulation of growth in PWN infected plants has been previously investigated in needle tissues of *P. massoniana*^21^. Plant hormone signalling genes were targeted by differentially expressed miRNAs, leading to the suppression of indole acetic acid and zeatin synthesis thus causing the inhibition of plant growth, but the role of the expressed miRNAs in regulating plant immune response was not addressed. In *P. pinaster*, sRNAs were reported to be involved in the regulation of embryo development^22^ and abiotic stress response^23^, but their function in biotic stress has not been described.

In this study, the regulatory roles of sRNAs in *P. pinaster*–PWN interaction during an early stage of infection (72 h post-inoculation, hpi) were investigated in PWN infected tissues (stem). While 105 pine differentially expressed (DE) miRNAs were found to be responsive to PWN and possibly regulating JA-response, ROS detoxification and terpenoid biosynthesis, only eight miRNAs were identified with predicted roles in PWN resistance. Our results suggest that post-transcriptional regulation of *RLK/RLP receptors* and *l-fucose synthase* by miRNAs might be a relevant mechanism involved in resistance to PWD. Furthermore, investigation of possible bidirectional trans-kingdom RNA silencing revealed that silencing of the host plant genes by PWN miRNAs may promote virulence, while targeting of PWN genes by the plant miRNAs may have a role in *P. pinaster* resistance to PWD.

## Results

To identify sRNAs involved in *P. pinaster* response and resistance to PWN, an inoculation assay was performed with plants from a half-sib family characterized by Carrasquinho et al*.*^9^. Within this family, individuals may present resistant or susceptible phenotypes after PWN inoculation, as previously described^9^. Sample collection from the stem of inoculated plants was performed at 72 hpi. After sampling, symptoms were observed weekly and plants were classified on a scale of 0 (no visible symptoms) to 4 (more than 75% of brown/wilted needles)^13^ (Table 1). After 210 days post-inoculation (dpi), 28% of the plants remained healthy (level 0) and were considered resistant, while 72% of the plants showed symptoms and were considered susceptible. The susceptible plants selected for RNA-seq were the first four plants showing a level 4 of symptoms in the symptoms scale. Symptoms evaluation and progression along the experiment have been previously detailed in Modesto et al*.*^13^.

**Table 1:** Symptoms’ progression in selected timepoints.

Symptoms	Days post inoculation (dpi)					
	14 dpi (%)	21 dpi (%)	35 dpi (%)	42 dpi (%)	105 dpi (%)	210 dpi (%)
0	83	72	44	44	28	28
1	11	17	28	22	22	17
2	6	11	11	0	0	5
3	0	0	6	11	0	0
4	0	0	11	23	50	50

Symptoms were evaluated weekly for 210 days post-inoculation (dpi) and registered according to a five-level scale based on percentage of brown/wilted needles: 0—0%; 1—1 to 25%; 2—26 to 50%; 3—51 to 75%; 4—7 to 100%.

### Small RNAs sequencing and identification

Small RNA libraries were sequenced for four susceptible, five resistant, and four control individuals. Small RNA sequencing data yielded approximately 23–40 million reads per sample, with sizes ranging between 18 and 50 bp. Since the nematode infects and migrates through stem tissues, and these tissues have been harvested during sampling, reads were mapped to both *Pinus taeda*^24^ and PWN genomes^25^. An average of 97% mapped reads was obtained, from which 99.5% mapped to the *P. taeda* genome, and 0.5% mapped to the PWN genome (Supplementary Table S1). Reads mapping to different genomes were analysed separately.

**Figure 1:**
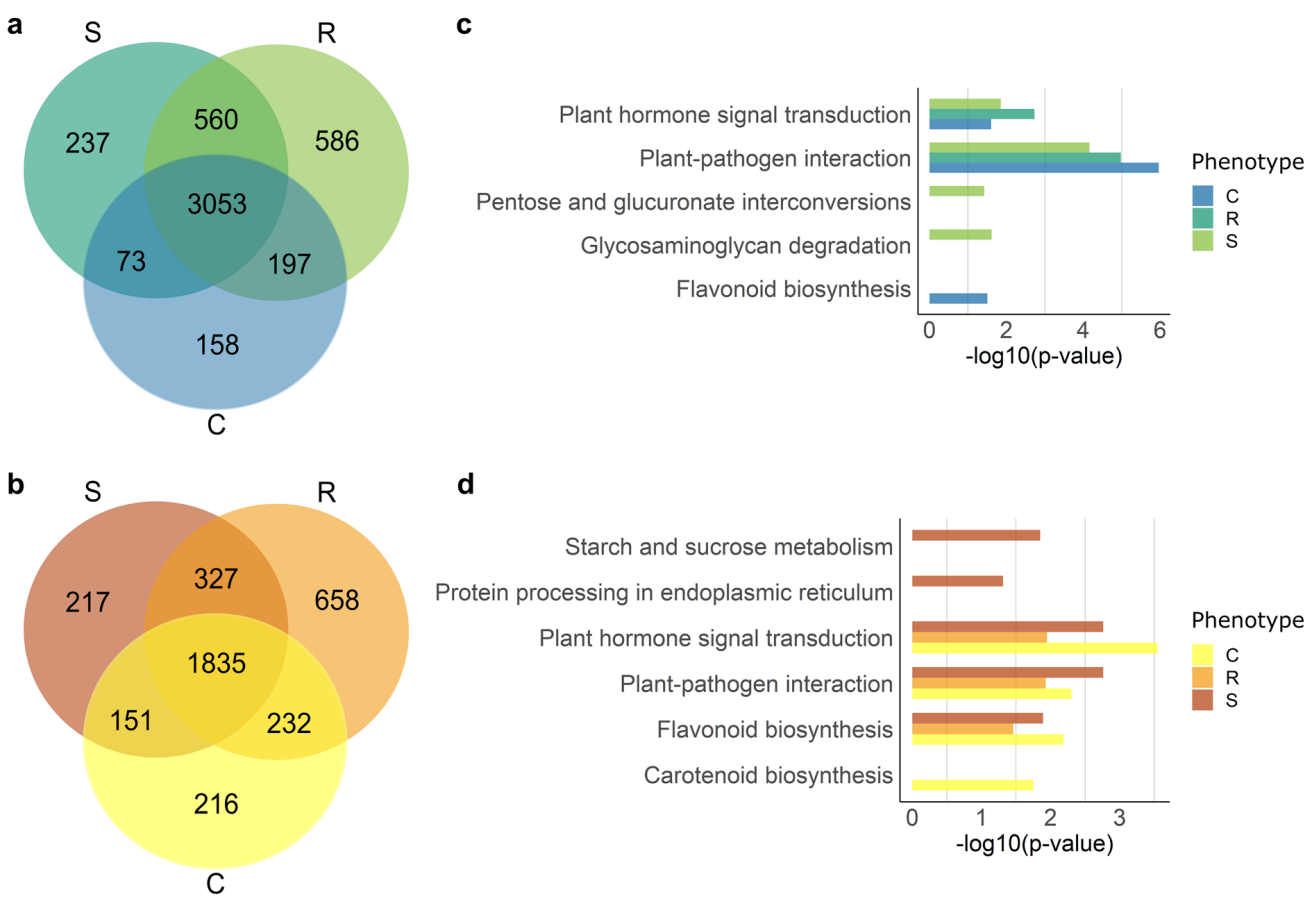
Number of expressed *Pinus pinaster* miRNAs (**a**) and tasiRNAs (**b**) in susceptible (S), resistant (R), and control (C) samples. Pathway enrichment analysis^26,27^ of predicted target genes of the expressed miRNAs (**c**) and tasiRNAs (**d**). The x-axis represents the significance of pathway enrichment (− log10 of corrected *p*-values) (**c,d**). Venn diagrams were generated online (https://bioinformatics.psb.ugent.be/webtools/Venn/) and edited with Inkscape 1.1 (https://inkscape.org/). Bar plots were generated with R 4.1.0 (https://cran.r-project.org/) ggplot2 package (https://ggplot2.tidyverse.org/).

An average of 18 million *P. pinaster* reads was retained per sample after initial filtering, with sizes between 18 and 26 nucleotides (Supplementary Table S1). This corresponds to 49–69% of the reads that mapped to the *P. taeda* genome, and most were 21 nt (≈ 50%) (Supplementary Fig. 1). Reads were analysed to identify conserved miRNAs, novel miRNAs, and trans-acting sRNAs (tasiRNAs). A total of 4984 miRNAs were identified in all samples (Fig. 1a, Supplementary Table S2), from which 850 were novel (Table 2). The conserved miRNAs belonged to 184 different families. A total of 3636 tasiRNAs were identified in all samples (Fig. 1b). A large part of the miRNAs (63%) and the tasiRNAs (50%) were expressed in all samples (Fig. 1).

**Table 2:** Numbers of small RNAs detected in *P. pinaster* and PWN, *B. xylophilus*. Values for susceptible, resistant and control samples represent the mean of the biological replicates.

	*Pinus pinaster*				*Bursaphelencus xylophilus*		
	Total	Susceptible	Resistant	Control	Total	Susceptible	Resistant
Conserved miRNA families	184	143 (± 2)	144 (± 2)	137 (± 3)	195	93 (± 22)	123 (± 18)
Conserved miRNA members	3506	3079 (± 75)	3213 (± 223)	2725 (± 234)	908	329 (± 91)	466 (± 88)
Novel miRNAs	850	506 (± 24)	529 (± 30)	447 (± 35)	78	41 (± 6)	48 (± 7)
Total miRNAs	4356	3586 (± 93)	3743 (± 251)	3173 (± 269)	986	369 (± 96)	514 (± 95)
tasiRNAs	3636	2070 (± 65)	2314 (± 194)	1967 (± 134)	–	–	–

For PWN originating sequences, an average of 100,000 reads with sizes between 18 and 26 nucleotides were retained per sample (Supplementary Table S1). This corresponds to 51–69% of the reads that mapped to the *B. xylophilus* genome and most of them were 21nt (≈ 52%; Supplementary Fig. 1b). Filtered reads were subsequently analysed to identify conserved miRNAs and novel miRNAs. A total of 986 miRNAs were identified in all samples (Table 2, Supplementary Table S3), from which 78 were novel. The conserved miRNAs belonged to 195 different families.

### *P. pinaster* miRNAs responsive to PWN and their target genes

**Figure 2:**
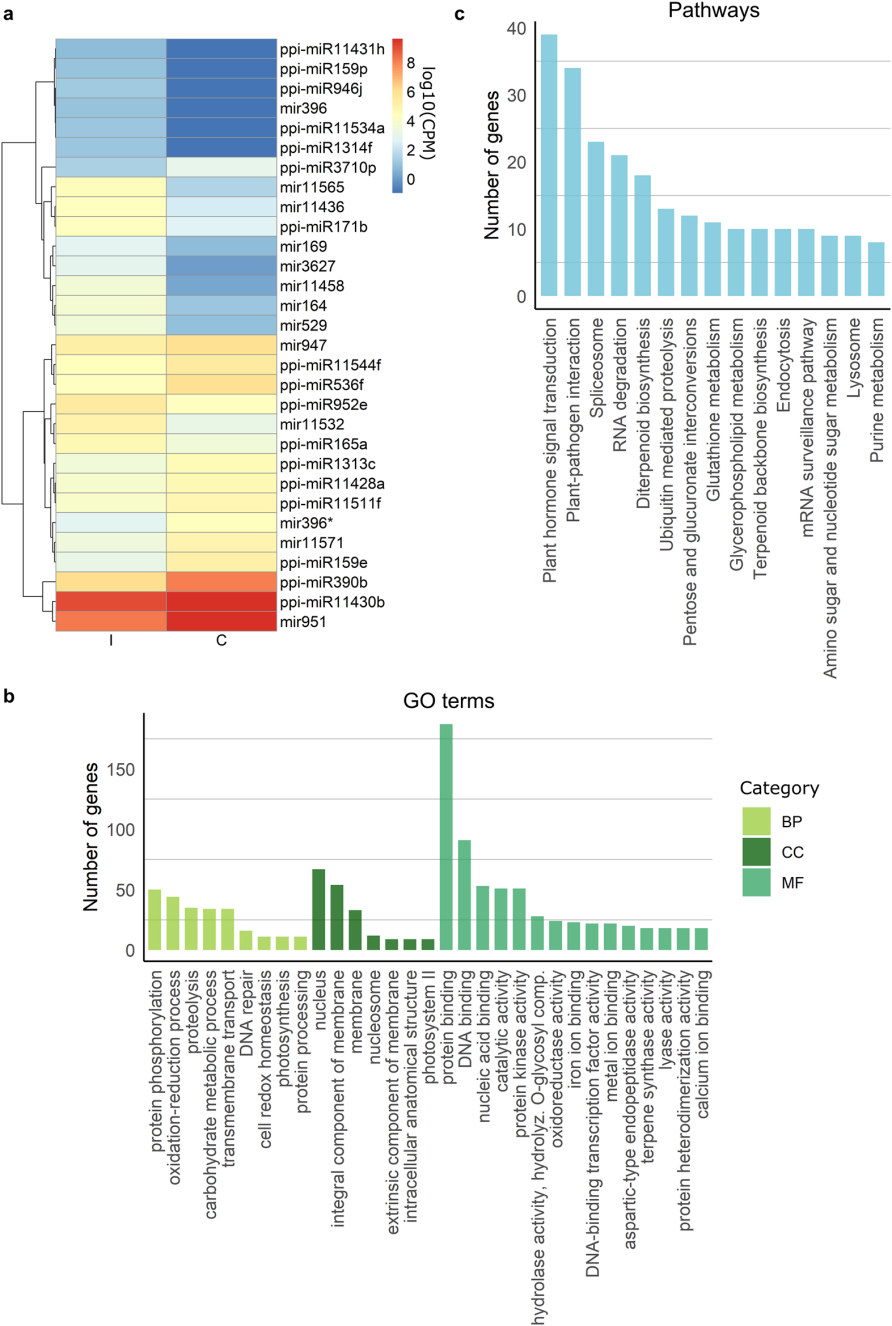
MiRNAs differentially expressed between inoculated (I) and control plants (C) and functional analysis of their target genes. (**a**) Average expression [log10(CPM)] for each conserved DE miRNA family, except for families where the miRNAs presented opposite expression patterns to each other, in which case isoform expression is represented. (**b**) GO terms and (**c**) pathways most represented in *P. pinaster* predicted target genes for the DE miRNAs. The y-axis represents the number of genes within each KEGG pathway^26,27^ or GO term. *BP* biological process, *CC* cellular component, *MF* molecular function. Plots were generated with R 4.1.0 (https://cran.r-project.org/) pheatmap package (https://cran.r-project.org/web/packages/pheatmap/) and ggplot2 package (https://ggplot2.tidyverse.org/). Inkscape 1.1 (https://inkscape.org/) was used to assemble the final figure.

Differential expression analysis between inoculated and control plants revealed 105 DE miRNAs (adjusted *p*-value ≤ 0.05; Supplementary Table S2), from which 79 were upregulated and 26 were downregulated. The DE miRNAs included 86 conserved ones, from 29 families (Fig. 2a). Some of these families had one single isoform differentially expressed (e.g. miR11428, miR11430), while 18 had two to 16 (miR529) isoforms (Supplementary Table S2). The mean expression for each family is shown in Fig. 2a.

To explore the putative function of the DE miRNAs, their target genes were predicted using psRNATarget and the transcriptome. Taking advantage of the transcriptomics data available for the same samples^13^, the analysis of negative correlations of gene expression levels between miRNAs and mRNAs was performed. In this way, it was possible to identify 1682 target genes (Pearson’s correlation R ≤  − 0.65; Supplementary Table S4).

After redundancy reduction, 184 GO terms were attributed to target genes (Supplementary Table S5). Doing a gene set enrichment analysis, only the biological process (BP) terms macromolecule modification and response to stimulus were significantly enriched (*p* ≤ 0.05). Within the most represented GO terms (Fig. 2b) were the BPs oxidation–reduction process and cell redox homeostasis, the cellular components (CCs) nucleus and integral component of membrane, and the molecular functions (MFs) DNA binding, protein kinase activity, and terpene synthase activity. Protein phosphorylation and protein binding were also highly represented in the analysis.

Regarding KEGG annotation^26,27^, 71 pathways were assigned to target genes (Supplementary Table S5). Plant hormone signal transduction was significantly enriched (*p* ≤ 0.05) in the targets of the DE miRNAs. The most represented pathways included plant–pathogen interaction, diterpenoid biosynthesis, and terpenoid backbone biosynthesis (Fig. 2c). Within the pathway plant hormone signal transduction were several jasmonate responsive genes, such as *JAZ/Tify* and *MYC4* transcription factors. The miRNAs targeting these genes were downregulated after inoculation (Table 3), suggesting an activation of the JA pathway. Plant–pathogen interaction genes, such as *WRKY* transcription factors, disease resistance proteins (*RLPs/RLKs*), and calcium-dependent protein kinase *CPK28*, were targeted by upregulated miRNAs (Table 3). Among the target genes, it was also possible to identify *terpene synthase* genes, such as *bifunctional abietadiene synthase* (*AS*) and *bifunctional levopimaradiene synthase* (*LPS*) (Table 3). Genes involved in detoxification of ROS were targeted by several upregulated miRNAs, including *peroxiredoxins*, *superoxide dismutase* (*MSD1*), or *thioredoxin* (Table 3).

**Table 3:** Selected differential expressed *P. pinaster* miRNAs and predicted target genes.

miRNA	Expression pattern	Log2FC	Target ID	Target annotation	GO terms/pathways
**DE miRNAs inoculated vs control**					
ppi-miR166f	Downregulated	− 0.623	unigene8322	Protein TIFY 6B-like	Plant hormone signal transduction
			unigene942	Protein TIFY 6B	Plant hormone signal transduction
ppi-miR947e	Downregulated	− 1.214	unigene105220	Protein TIFY 10A	Plant hormone signal transduction
ppi-miRnovel43f	Downregulated	− 1.541	unigene26097	Transcription factor MYC4-like	Plant hormone signal transduction
ppi-miR390b	Downregulated	− 2.065	unigene3146	Nematode resistance protein-like HSPRO1	Defence response
ppi-miR11565a-i	Upregulated	3.734 (± 1.83)	isotig42180	WRKY transcription factor 20	Plant–pathogen interaction
			unigene650	WRKY transcription factor 44	Plant–pathogen interaction
ppi-miRnovel816	Upregulated	1.754	isotig42166	Calcium-dependent protein kinase 28 (CPK28)	Plant–pathogen interaction
ppi-miR11565h	Upregulated	5.948	unigene12702	Disease resistance RPP13-like protein 4	Plant–pathogen interaction
ppi-miR11458e	Upregulated	6.313	isotig49219	Disease resistance protein At1g61300	Plant–pathogen interaction
ppi-miR11458f	Upregulated	5.155	unigene57660	Disease resistance protein RPS2	Plant–pathogen interaction
ppi-miR3627u	Upregulated	5.053	isotig51344	Disease resistance protein RPS2-like	Plant–pathogen interaction
ppi-miR529l	Upregulated	5.121	unigene116482	Probable RLK	Plant–pathogen interaction
ppi-miR946j	Upregulated	5.178	isotig75044	Disease resistance RPP13-like protein 4	Plant–pathogen interaction
ppi-miR396j	Upregulated	4.923	unigene75931	Disease resistance protein At4g27190-like	Plant–pathogen interaction
			isotig42452	Bifunctional levopimaradiene synthase (LPS)	Diterpenoid biosynthesis
Novel_1871	Upregulated	1.887	unigene31062	Bifunctional abietadiene synthase (AS)	Diterpenoid biosynthesis
ppi-miR11436b, f-k, m	Upregulated	2.428 (± 1.86)	unigene2998	Bifunctional abietadiene synthase (AS)	Diterpenoid biosynthesis
	Upregulated		unigene9633	Bifunctional abietadiene synthase (AS)	Diterpenoid biosynthesis
ppi-miR11436b, f-m	Upregulated	2.754 (± 2.00)	isotig44195	4-Hydroxy-3-methylbut-2-enyl diphosphate reductase	Terpenoid backbone biosynthesis
ppi-miRnovel1251	Upregulated	4.890	unigene97227	Bifunctional levopimaradiene synthase (LPS)	Monoterpenoid biosynthesis
ppi-miR3627s	Upregulated	5.761	isotig56835	Short-chain dehydrogenase/reductase 2b-like	Monoterpenoid biosynthesis
ppi-miR11436b, f, g, j, k, m	Upregulated	2.725 (± 2.11)	isotig34808	Peroxiredoxin Q	Cell redox homeostasis
ppi-miR1314f	Upregulated	5.123	isotig25066	Peroxiredoxin-2E	Cell redox homeostasis
ppi-miR3627l	Upregulated	3.980	isotig25066	Peroxiredoxin-2E	Cell redox homeostasis
ppi-miR529c, y	Upregulated	4.383 (± 2.91)	isotig12834	Thioredoxin F-type	Cell redox homeostasis
**DE miRNAs resistance vs susceptible**					
ppi-miR166h	R > S	6.418	unigene67614	Putative RLK	Protein serine/threonine kinase activity
ppi-miR951f	R < S	− 1.329	unigene93826	Putative RLK	Protein serine/threonine kinase activity
			unigene5558	Putative RLK	Protein kinase activity
ppi-miR947f	R > S	5.163	isotig45349	GDP-L-fucose synthase 2	Amino sugar and nucleotide sugar metabolism
Novel_110	R < S	− 1.550	isotig51371	Protein COBRA-like	Cellulose microfibril organization
			unigene925	Ninja family protein	Signal transduction
ppi-miR3627m	R < S	− 3.661	unigene82871	Short-chain dehydrogenase reductase 2a-like	Oxidoreductase activity

Five Pfam protein domains were enriched in *P. pinaster* DE miRNAs predicted target genes (*p* ≤ 0.05; Supplementary Table S5), including F-box domain, which mediates protein–protein interactions, and SBP domain, found in transcription factors.

RT-qPCR analysis of five DE miRNAs showed similar expression trends as the small RNA-seq results (Pearson’s correlation R = 0.77, *p* = 0.009; Supplementary Fig. 2). For each of these miRNAs, RT-qPCR analysis was performed for one predicted target gene and a strong positive correlation was found between RT-qPCR and RNA-seq results (Pearson’s correlation R = 0.97, *p* = 1.8e−06; Supplementary Fig. S2). A correlation analysis was also made between the RT-qPCR values of the miRNAs and respective predicted target gene. For two pairs of miRNA-target gene a high negative correlation value, although not significant, was obtained (miRNovel-RPP13 Pearson’s R =  − 0.78; miR11436b-RLK3 Pearson’s R =  − 0.61), while for the remaining pairs low or positive correlation coefficients were obtained (Supplementary Table S6).

### miRNAs associated with PWN resistance and their target genes

**Figure 3:**
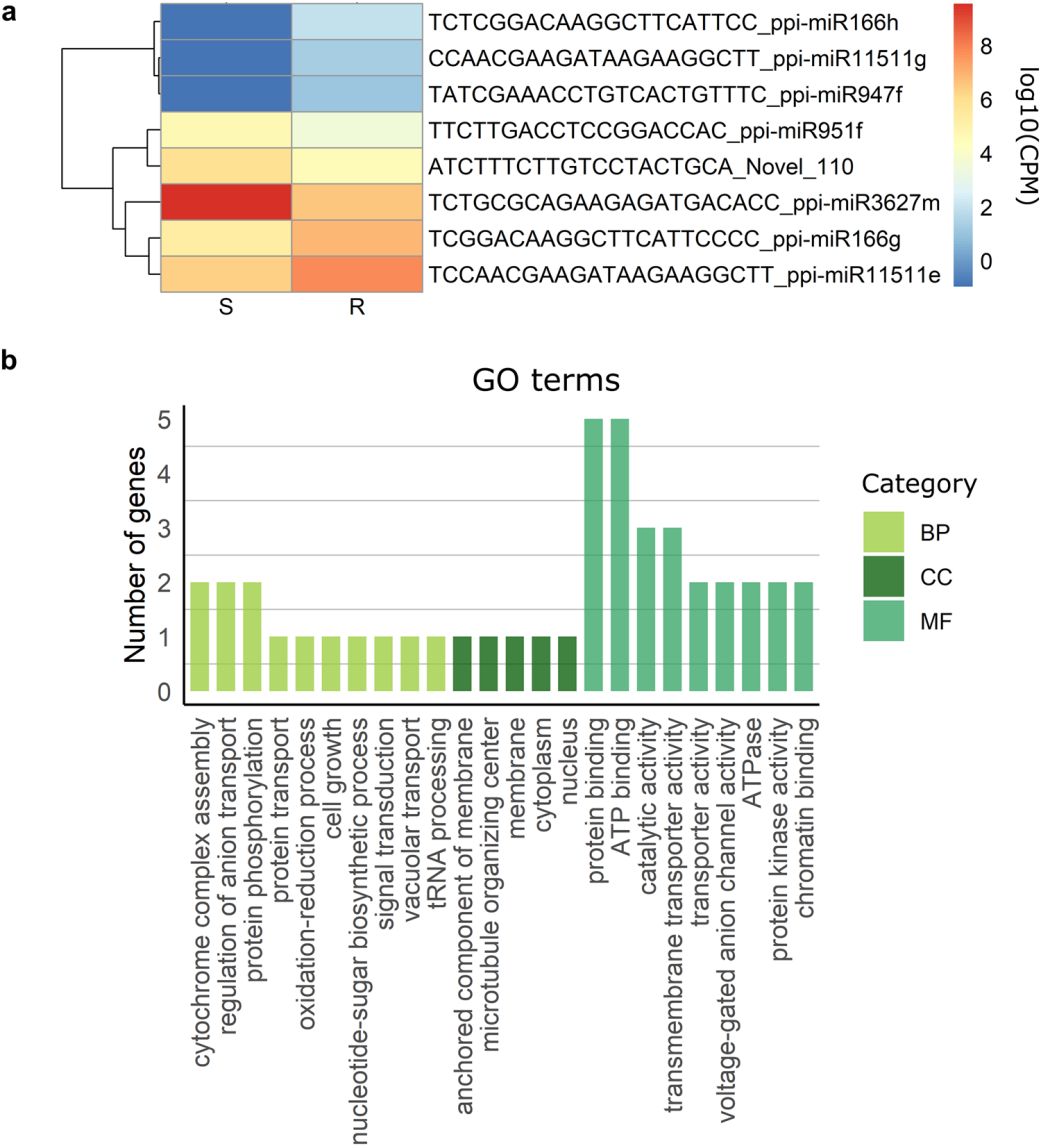
MiRNAs differentially expressed between resistant (R) and susceptible (S) plants (**a**) and functional analysis of their target genes (**b**). (**a**) The heatmap represents average log10(CPM) values for each miRNA. (**b**) GO terms represented in the predicted target genes for the DE miRNAs. The y-axis represents the number of genes within each GO terms. *BP* biological process, *CC* cellular component, *MF* molecular function. Plots were generated with R 4.1.0 (https://cran.r-project.org/) pheatmap package (https://cran.r-project.org/web/packages/pheatmap/) and ggplot2 package (https://ggplot2.tidyverse.org/). Inkscape 1.1 (https://inkscape.org/) was used to assemble the final figure.

To identify miRNAs that may have a role in resistance to PWN, resistant and susceptible samples were compared, revealing eight miRNAs DE between these two groups (adjusted *p*-value ≤ 0.05; Fig. 3a). From these, seven were conserved miRNAs, corresponding to five families (Fig. 3a): miR166, miR947, miR951, miR3627, and miR11511. These families were also, as previously mentioned, differentially expressed after inoculation (Fig. 2a), although the isoforms detected as significantly differentially expressed were distinct (Supplementary Table S2).

Negative correlations of expression levels between miRNAs and predicted targets^13^, led to the detection of 37 target genes (Pearson’s correlation R ≤  − 0.65; Supplementary Table S7). After redundancy reduction, 24 GO terms were attributed to these target genes (Fig. 3b), including the BPs oxidation–reduction process, signal transduction, and the MF protein kinase activity. KEGG pathway terms^26,27^ were attributed only to six of the target genes and included endocytosis, phagosome, amino sugar and nucleotide sugar metabolism, proteasome, lysine degradation, and pyrimidine metabolism (Supplementary Table S7).

Within the target genes, it was possible to identify three *RLK*s (Table 3). One of these genes was targeted by miR166h, which was expressed at higher levels in resistant plants, while the other two were targeted by miR951f, which were both expressed at higher levels in susceptible plants. *GDP-L-fucose synthase 2* was targeted by miR947f, which was more expressed in resistant plants (Table 3). The miRNAs miR3627m and Novel_110, which showed increased expression in susceptible plants, targeted a gene with oxidoreductase activity and a COBRA protein-encoding gene, involved in cellulose deposition, respectively. Novel_110 also targeted a gene encoding for a Ninja family protein, which negatively regulates the JA defence response (Table 3).

### Differentially expressed miRNAs and tasiRNA production

Several of the DE miRNAs here detected have been previously identified as leading to the production of tasiRNAs in *Picea abies*^28^. These miRNAs targeted *NB-LRR* resistance genes, non-coding RNAs, and genes of unknown function. TasiRNAs commonly originate also from genes of the *pentatricopeptide repeat-containing protein* (*PPR*) family^29^. Here, it was possible to identify targets with similar annotations for five miRNAs of the families miR947, miR3627, and miR11532 (Supplementary Table S8). Four of these transcripts were indeed predicted to originate sequences of tasiRNAs in the analysed *P. pinaster* samples. Three of these transcripts encode NB-LRR resistance proteins, targets of the miR11532 family, and one encodes a gene of unknown function, targeted by miR947f (Supplementary Table S8). Predicted targets of tasiRNAs included genes involved in plant hormone signal transduction, plant-pathogen interaction, and flavonoid biosynthesis pathways in all three groups of samples (Fig. 1d).

### Investigation of miRNA mediated trans-kingdom interaction

As interactions between the miRNAs of parasites and the transcripts of their host plants have been previously reported^14–16^, we searched for possible targets of PWN miRNAs in the *P. pinaster* transcriptome. Only predicted targets with an expression that correlated negatively with the expression of the PWN miRNAs were maintained. Remarkably, this led to the detection of 2515 target genes (Pearson’s correlation R ≤  − 0.65; Supplementary Table S9).

**Figure 4:**
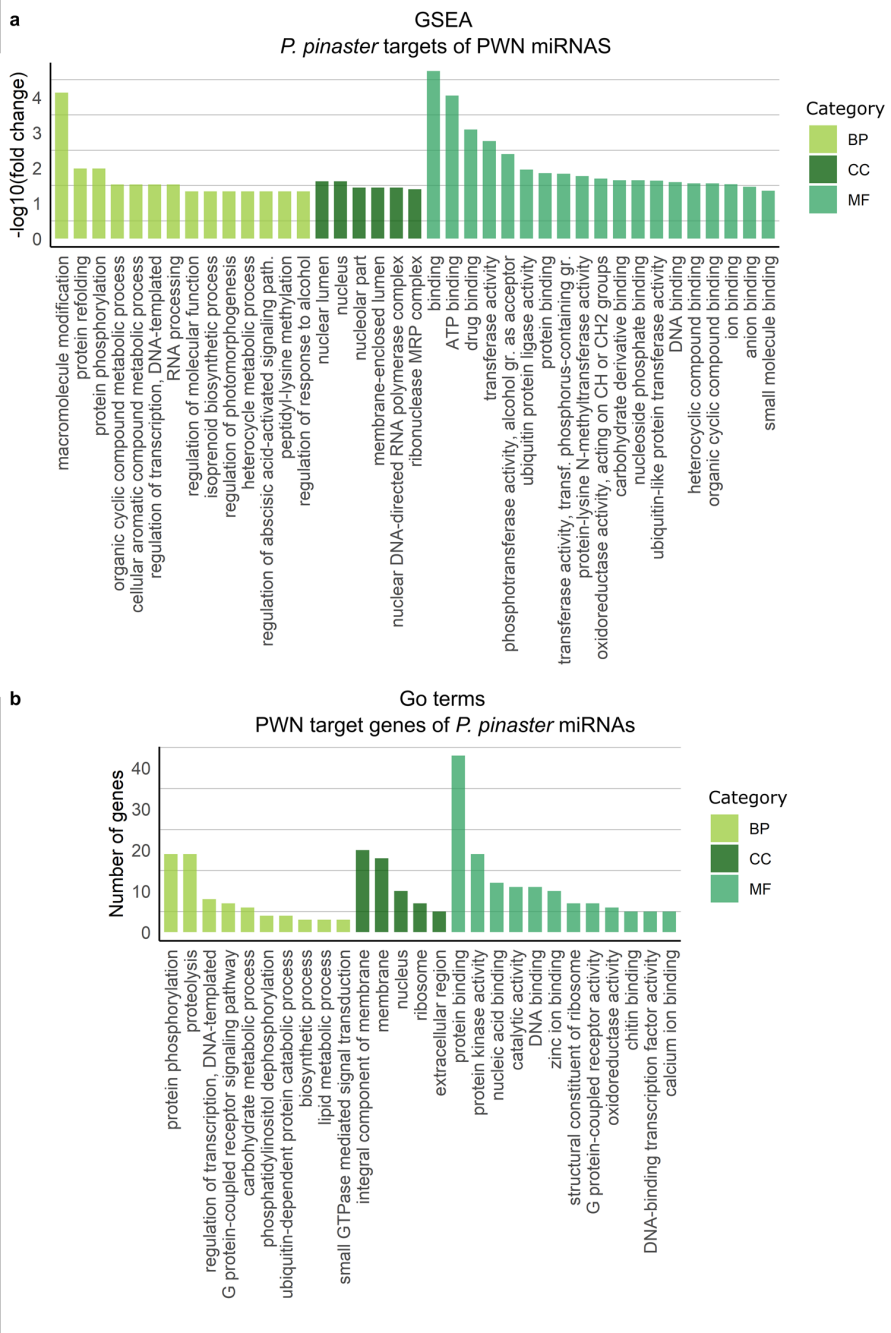
Functional analysis of the predicted target genes of miRNAs possibly involved in trans-kingdom interaction. (**a**) Gene set enrichment analysis (GSEA) of GO terms represented in *P. pinaster* predicted targets for PWN miRNAs. The y-axis represents the significance of pathway enrichment (− log10 of corrected *p*-values). (**b**) GO terms represented in PWN predicted target genes for *P. pinaster* miRNAs. The y-axis represents the number of genes within each GO terms. Plots were generated with R 4.1.0 (https://cran.r-project.org/) ggplot2 package (https://ggplot2.tidyverse.org/). Inkscape 1.1 (https://inkscape.org/) was used to assemble the final figure.

Gene set enrichment analysis revealed 39 enriched GO terms after redundancy reduction (Supplementary Table S10) and included general BPs like protein refolding, protein phosphorylation, and RNA processing, as well as MFs such as ATP binding, transferase activity, and protein binding (Fig. 4a). On the other hand, some of the target genes seem to be involved in BPs more directly connected to plant defence response, such as isoprenoid biosynthetic process and regulation of abscisic acid-activated signalling pathway (Fig. 4a). The most represented pathways included spliceosome, ribosome, and mRNA surveillance pathway, but also plant hormone signal transduction, terpenoid backbone biosynthesis, and MAPK signalling pathway (Supplemental Table S9). The Pfam protein kinase domain was also significantly enriched (Supplemental Table S10).

The use of degradome data to further support the targeting of *P. pinaster* transcripts by PWN miRNAs allowed for the identification of 116 target regions (Supplementary Table S11). When applying stricter filters, such as selecting only regions with a score higher than three (more than one degradome read in the position, but lower coverage than the average of the corresponding transcript) or than two (coverage on the site is higher than the average of the corresponding transcript), 60 and 41 target regions, respectively, were still retained. From the 116 target regions, only 12 were predicted to be also targeted by *P. pinaster* sRNAs (Supplementary Table S12).

Target genes identified in the degradome have GO annotations ranging from photosynthesis, structural constituent of ribosome, and ATP binding (Supplementary Table S13) to defence response to fungus and oxidation–reduction process. Target genes with known roles in plant defence response included *thaumatin-like proteins*, *PR-4*, *RLK*, genes involved in flavonoid biosynthesis (*chalcone synthase 1* and *caffeoyl-CoA O-methyltransferase*), and *thioredoxin H4-1*, involved in cell redox homeostasis (Supplementary Table S11).

Trans-kingdom interactions through sRNAs have been described in both directions, this is, sRNAs from plants may also target pathogens or parasites genes^19,20^. Therefore, targets for *P. pinaster* miRNAs DE between susceptible and resistant plants were predicted in the PWN transcriptome and led to the identification of 552 target regions in 487 PWN genes (Supplementary Table S14). Analysis of the targets’ GO annotations and pathways (Fig. 4b, Supplementary Table S15) reveal that *P. pinaster* miRNAs may target genes important for PWN response to stimuli (e.g. MF protein kinase activity; BP G protein-coupled receptor signalling pathway), transcriptional response (e.g. BP regulation of transcription, DNA-templated; pathways spliceosome and ribosome), detoxification of plant xenobiotic compounds (e.g. MF oxidoreductase activity; pathway metabolism of xenobiotics by cytochrome P450), and digestion of plant tissues (e.g. BPs proteolysis and carbohydrate metabolic process; pathways lysosome or protein digestion and absorption).

## Discussion

The importance of miRNAs in plant response to biotic and abiotic stresses has been repeatedly demonstrated in the last years^16,30^. Several studies have shown an important regulatory role of miRNAs in plant response to parasitic nematodes^31^. However, the role of miRNAs in the defence response to PWN has not been previously reported and few studies focussed on defence response in conifer species^32,33^. The expression of miRNAs after PWN inoculation was previously analysed in *P. massoniana*^21^, but this analysis was made in needles to study regulation of plant growth and no insights are currently available regarding the post-transcriptional regulation of genes or pathways possibly involved in defence response against PWN. In this study, we investigated the role of miRNAs in the regulation of *P. pinaster* defence response to PWN inoculation, explored their involvement in resistance to PWD and, finally, identified miRNAs that may have an important role in sRNA mediated trans-kingdom interaction.

MiRNAs can regulate gene expression by mRNA cleavage or translation inhibition^34^. In plants, the most common mechanism is target cleavage^34^, in which case the expression of a miRNA and its respective targets is expected to correlate negatively. Taking this into account, we combined the miRNA data here obtained with mRNA expression data of the same samples previously described in Modesto et al*.*^13^. This approach allowed us to narrow down significantly an extensive list of possible gene targets and increase the reliability of the final targets list. RT-qPCR results supported a strong negative correlation between the expression of two of the five miRNA-target gene pairs tested. For the remaining pairs, it is possible that the expression of other predicted targets not tested here may show high negative correlation, but also alternative miRNA regulatory mechanisms beyond mRNA target cleavage should not be excluded. A more extensive testing would be necessary to have additional insights into the miRNA-target regulatory relation.

Analysing the DE miRNAs between inoculated and control samples, it was possible to identify a set of *P. pinaster* miRNAs involved in response to PWN inoculation. Several of the conserved miRNAs families have been described as involved in response to root-knot nematode or cyst nematode in Arabidopsis^35^, cotton^36^, tomato^37^ and soybean^31,38^, including miR159, miR390, miR396, miR164, miR166, and miR3627. In such interactions, the expression of these miRNAs has been associated with cyst or gall formation. As PWN life strategy is different from sedentary nematodes, and their survival does not depend on the formation of those specialised feeding structures, the role of these miRNAs in response to PWN is likely different. The predicted targets having a negatively correlated expression with these miRNAs were, in fact, distinct from what is described in the literature^31,35–38^. For instance, while several MYB transcription factors were predicted for miR159, as described for other nematode-plant interactions^31^, the expression of the miRNAs and respective target transcripts were not negatively correlated. This suggests that miR159, as well as the other mentioned conserved miRNAs, regulate different genes and pathways in *P. pinaster* response to PWN, when compared to the response to sedentary nematodes in angiosperms.

Several of the identified PWN responsive miRNAs were previously described as involved in *P. taeda* response to fusiform rust^32^. This includes the conserved families miR159, miR166, miR171, miR390 or miR396, and Pinaceae specific conserved families miR946, miR947, miR951, and miR952. However, the *P. pinaster* targets here predicted for these miRNAs were different from *P. taeda* targets or were not negatively correlated with the corresponding miRNA expression. Therefore, although the miRNAs involved in response to pathogens and parasite nematodes seem to be partly conserved, both between angiosperms and gymnosperms, as well as between these two closely related *Pinus* species, they may regulate different defence mechanisms. The defence mechanisms induced by biotrophic pathogens, such as fusiform rust fungus or sedentary nematodes, and migratory nematodes or herbivore insects are often described as antagonistic^39^.

Part of the miRNA families here detected as differentially expressed after PWN inoculation were also responsive to drought stress in *P. pinaster*^23^ (miR159, miR164, miR166, miR169, miR396, miR529, miR1313, miR3627, miRnovel578), suggesting these families may have a role in the regulation of stress responses in general.

In this work, we showed that some of the pathways previously pointed out as relevant for *P. pinaster* response to PWN inoculation^12,13,40^ seem to be post-transcriptionally regulated by miRNAs. These include plant hormone signalling pathways, of which the JA response pathway is highlighted. The induction of JA immunity has been earlier reported in *P. pinaster* in response to PWN^12,13,40^ and associated with resistance^13^. Several miRNAs here observed to be responsive to PWN infection or associated with resistance (miR947 and miR951), belong to families previously described as responsive to methyl-jasmonate (MeJA) treatment in *Taxus chinensis* (miR164, miR169, miR390, miR396)^41^ or *Pinus sylvestris* (miR946, miR947, miR951, miR952)^33^. Additionally, miRNAs of the families miR166 and miR947 seem to target *JAZ/Tify* transcription factors, which repress JA response, while the novel miRNA miRnovel43f seem to target *MYC4* transcription factor, which induces JA response^42^. The downregulation of miR166, miR947, and miRnovel43f suggests their expression is inhibited by higher levels of JA^13^, inducing the expression of their targets. The DE of the miRNA Novel_110 and respective target, the JA defence response regulator *NINJA*, between resistant and susceptible plants indicates that this hormone has also an important role in *P. pinaster* resistance to PWN, as previously suggested^13^. JA immune response seems to be, therefore, tightly regulated during *P. pinaster* response to PWN, both at the transcriptional and post-transcriptional levels, and the results here obtain further support its important role in resistance to this nematode.

Several of the identified targets of DE miRNAs were *RLK*s or *RLP*s, involved in the activation of PTI. Noticeably, when comparing susceptible and resistant plants, the two miRNAs targeting different *RLK* genes had contrasting expression patterns, with miR166h more expressed in susceptible plants and miR951f more expressed in resistant plants. The different post-transcriptional regulation of the targeted *RLKs* in susceptible and resistant plants may lead to the activation of distinct defence pathways. A contrasting differential expression of RLK/RLP encoding genes in resistant and susceptible plants has been previously associated with *P. pinaster* resistance to PWN^13^.

ROS detoxification has been described as an important part of plant defence response^10,43^ and in particular in *Pinus* spp. response to PWN infection^12,13,44,45^. In this work, several genes involved in maintaining cell redox homeostasis, as *peroxiredoxins* and *thioredoxins*, seem to be regulated by miRNAs induced after PWN inoculation, supporting the importance of this mechanism in *P. pinaster* defence response to PWN. In susceptible plants, higher expression levels were observed for miR3627m, which targets a gene encoding for a protein with oxireductase activity, suggesting that susceptible plants might have lower ROS detoxification ability when compared to resistant plants. A better and more prolonged ROS detoxification was associated with PWN resistance in *P. massoniana*^44^, while higher expression of oxidative stress response genes was observed in resistant *P. thunbergii*^45^ and *P. pinaster*^13^.

Terpenoids are important compounds in *Pinus* spp. defence against several pests^46^. Multiple genes encoding enzymes involved in terpenoid biosynthesis pathways were targeted by DE miRNAs, highlighting the importance of these compounds in response to PWN. Increased expression of terpene synthases, including *AS* and *LPS* genes, has been previously reported in *P. pinaster*^13^ and *P. massoniana*^44^ response to PWN and associated with PWN resistance. Moreover, the products of two *P. massoniana* terpene synthases, α-pinene and longifolene, directly inhibited the survival rate of PWN in vitro^47^, reinforcing the importance of these compounds in plant response and resistance to PWN.

The role of l-fucose biosynthesis and protein fucosylation in plant defence response has been recently highlighted in Arabidopsis^48^. In Arabidopsis, fucosylation of RLKs/RLPs was found to be essential for the normal activation of PTI and ETI. Interestingly, miR947f, differentially expressed between susceptible and resistant plants, seem to target a *GDP-l-fucose synthase*. The post-transcriptional regulation of a *GDP-l-fucose synthase* points to a relevant role of fucosylation in achieving resistance to PWN. Additional studies may clarify if an earlier activation of this gene is detected in resistant plants prior to the 72 hpi for the fast activation of PTI upon inoculation.

In recent years, evidence for trans-kingdom transference of sRNAs has been accumulating^16,49^, including in host–pathogen and host-parasite interactions. In plants, examples of sRNA transfer between plant and pathogenic fungi or oomycetes have been reported^17,19,20,50^. For instance, *Botrytis cinerea* miRNAs targeted and silenced Arabidopsis transcripts with important roles in plant immunity, such as *MAPK*s and *WRKY* transcription factors^17,18^. Transference of miRNAs from plant to pathogen has also been reported^16,19,20^. *Gossypium hirsutum* miR166 and miR159 were transferred to the fungus *Verticillium dahlia*, targeting genes essential for the virulence of this fungus^20^. Furthermore, bidirectional sRNA transfer and trans-kingdom transcript cleavage was described in the interaction between the oomycete *Plasmopara viticola* and grapevine^19^. Therefore, sRNA transference between pathogens and plant hosts seems to be an important strategy both for plant defence and resistance, as well as for pathogen virulence. Here, we report several *P. pinaster* transcripts predicted as targets of PWN miRNAs. The silencing or downregulation of many of these target genes, such as transcriptional factors, RNA processing genes, ribosomal proteins or protein folding genes, may negatively affect the plant cell transcriptional response, as well as protein synthesis and correct assembly. On the other hand, several of the predicted targets are directly involved in plant immune response, such as genes involved in plant hormone signal transduction, terpenoid backbone biosynthesis, and MAPK signalling. The simultaneous targeting of genes important for protein synthesis, synthesis of toxic compounds, as well as early initiation and onset of the plant immune response, can affect the plant capacity to set a timely and appropriate defence response to PWN and therefore may be essential for the virulence of this nematode. The silencing of *P. pinaster* transcripts by PWN was supported by degradome data obtained from similar *P. pinaster* samples inoculated with the same PWN strain and collected at the same timepoint. Accordingly, it was possible to validate several *P. pinaster* targets using this approach. A very small number of the target sites predicted using degradome data were also predicted as target sites for *P. pinaster* miRNAs, supporting that the cleavage was guided by PWN miRNAs for most of the predicted targets.

In the opposite direction, the targeting of PWN genes by *P. pinaster* sRNAs, several interactions were also predicted. Contrasting with plants, post-transcriptional regulation in animals commonly involves the inhibition of translation of the targeted transcripts, and not their cleavage^51^. In this way, validating this interaction is not possible through degradome analysis. Nevertheless, analysing PWN genes targeted by *P. pinaster* miRNAs differentially expressed between resistant and susceptible plants may give us important information about resistance mechanisms. The miRNAs differentially expressed between resistant and susceptible plants were predicted to target several genes expressed in PWN pharyngeal gland cells and intestine^52^, several of which encode proteins previously detected in PWN secretome^53^. These genes are important for PWN evasion of plant defence response, PWN migration through plant tissues, and feeding. For instance, genes like *cytochrome P450* or *epoxide hydrolase* encode enzymes that degrade xenobiotic compounds produced by the plant host, allowing for the PWN to survive in the hostile environment. On the other hand, *peptidases* may be involved in the degradation of plant defence proteins and the digestion of plant tissues, which allows for migration throughout the plant and nutrients uptake, but can also be essential for embryogenesis and larval development^25^. Lysosomal enzymes may also play an important role in the digestion of ingested proteins in PWN, as it was observed in *C. elegans* intestine-specific secondary lysosomes^25^. In this way, the differential targeting of these genes by *P. pinaster* miRNAs in resistant and susceptible plants may affect PWN survival and development, contributing to the contrasting observed phenotypes.

Although no naturally occurring transference of sRNAs has been described in nematode–plant interactions, host-induced gene silencing (HIGS) has been shown to be an efficient method to manage these parasites^16,49^. This strategy involves the engineering of plant hosts to express RNA interference (RNAi)-inducing dsRNA that target and silence, in this case, nematode genes important for their growth, development or pathogenicity^54^. For instance, the transformation of potato plants (*Solanum tuberosum*) with an RNAi construct complementary to a root-knot nematode (*Meloidogyne chitwoodi*) effector gene increased plant resistance to this nematode^55^. In soybean, RNAi constructs targeting two genes potentially essential to root-knot nematode (*Meloidogyne incognita*) survival restricted greatly the number of galls formed in the plant roots^56^. Therefore, the uptake by the nematode of these dsRNAs or RNAi produced by the host conferred resistance in the transgenic plants. Although the process of sRNA translocation between organisms is not yet clear, sRNAs or sRNA-protein complexes seem to be more likely transported by extracellular vesicles^16,49^. Trans-kingdom RNA silencing can open new perspectives of fighting PWN through the development of HIGS, which was shown to be an ecological and efficient method for parasite management^16,49^.

In conclusion, this work provides new insights into the relevance of post-transcriptional regulation in *P. pinaster*–PWN interaction during the early stages of infection. The set of candidate miRNA-target nodes identified here represents an important foundation for future functional characterization studies in the context of PWD and PWN resistance. Furthermore, a possible role for trans-kingdom miRNA transfer and gene silencing was revealed, both for PWN parasitism and *P. pinaster* resistance. Although degradome analysis experimentally supported the silencing of *P. pinaster* genes by PWN miRNAs, further experimental work confirming the transference of miRNAs between organisms, the physical interaction between miRNA-target genes and subsequent gene silencing, would be of great relevance to better understand the significance of this bidirectional interaction in PWD and PWN resistance.

## Materials and methods

### Plant material and PWN inoculum

The *P. pinaster* half-sib family 440 was previously evaluated regarding the genetic effects on survival after PWN inoculation of 2-year-old plants^9^, showing a predicted survival mean of 15% (in a range of 6–23%). Seeds, provided by Dr. Isabel Carrasquinho (INIAV, Portugal), were collected from the mother tree 440, which is included in the reference population for PWD resistance^57^, located in “Herdade da Comporta” (38° 21′ 28.52′′ N, 8° 45′ 49.89′′ W) in southern Portugal. The necessary permissions were obtained for the collection and use of the seeds. Relevant institutional, national, and international guidelines for plant material collection and experimental work were followed. Four-year-old plants, germinated from the collected seeds, were maintained in 4 L pots in a greenhouse and placed according to a completely randomized experimental design.

*B. xylophilus* isolate Bx013.003^9,13,40^, obtained from an infected *P. pinaster* tree in a field in central Portugal (39° 43′ 33.8′′ N, 9° 01′ 55.7′′ W) and included in INIAV’s Nematology Laboratory collection (Oeiras, Portugal), was used for the inoculation assay. The sequence of the ITS region of this isolate is available in GenBank (NCBI) with the accession number MF611984.1. PWNs were maintained in culture at 25 ± 1 °C on a non-sporulating *Botrytis cinerea* strain grown on autoclaved barley grains. Previous to inoculation, nematodes were allowed to grow on sterilized wood and then isolated using the “tray” method^58^. Nematodes were suspended in water at a concentration of 1000 PWN/mL.

### Inoculation with PWN, sample collection, and evaluation of symptoms

Twenty-three plants were inoculated in September 2016 using the method described in Futai and Furuno^59^. Eighteen plants were inoculated with a suspension of 500 nematodes at mixed developmental stages, while five control plants were inoculated with sterile water. The inoculum was pipetted into a small longitudinal wound made in the main stem with a sterile scalpel below the apical shoot region^13^. Stem samples of approximately 5 cm, including the inoculation zone, were collected 72 hpi and immediately frozen in liquid nitrogen. The remaining part of each plant, below the inoculation zone, was kept in the greenhouse and observed weekly for 210 days. The progression of symptoms was registered by classifying the plants on a scale of 0 (no visible symptoms) to 4 (more than 75% of needles brown/wilted) in each observation point (Table 1). The first symptoms were observed 14 days post-inoculation (dpi) and evolved progressively until the end of the experiment. Plants that presented symptoms (1–4 on the scale) were classified as susceptible, while plants that did not present any symptoms (0 on the scale) were classified as resistant. This classification was based on external symptoms only and it is unknown if PWN multiplication was impaired in plants without symptoms, showing true resistance, or if plants maintained a healthy phenotype despite PWN multiplication, showing tolerance instead^60^.

### RNA extraction and sRNA sequencing

Five resistant, four susceptible, and four control plants were selected for sequencing. The four chosen susceptible plants were the first presenting the maximum level of symptoms (level 4). Total RNA, including the small RNA fraction, was extracted from stem samples after debarking using the method described in Provost et al*.*^61^. RNA and miRNA concentrations were determined using Qubit™ 4 Fluorometer (Thermo Fisher Scientific, Waltham, MA USA) with the RNA BR Assay Kit and miRNA Assay Kit. RNA integrity was checked with LabChip GX (PerkinElmer, Hopkinton, MA USA). Libraries were prepared with the Illumina TruSeq Small mRNA protocol and sequenced on Illumina HiSeq 2500 (Fasteris, Switzerland), providing 50 bp single-end reads. Each sample was run in two independent lanes.

### Identification of small RNAs and differential expression analysis

The quality of the small RNA-seq data was checked using FastQC v 0.11.4^62^. Adapter and quality trimming was performed using Trimmomatic^63^. As samples included *P. pinaster* and PWN RNA, to be able to distinguish between sequences originating from each organism, reads were mapped to *Pinus taeda*^24^ and PWN^25^ genomes using BWA alignment software v0.7.17 (BWA-backtrack algorithm)^64^. Separate fastq files were prepared with reads originating from plant or nematode.

Reads were then processed with the sRNA analysis pipeline miRPursuit^65^. In an initial step, data was filtered to remove t/rRNAs, low complexity reads, reads with an absolute abundance ≤ 5, and reads outside the range of 18–26 nucleotides. For *P. pinaster* originating reads, conserved miRNAs were identified by comparing the reads with mature plant miRNAs from the miRBase v22 database (www.mirbase.org), allowing for up to 3 mismatches. Novel miRNAs and tasiRNAs were predicted using default parameters. For PWN, conserved reads were annotated by comparing with previously described PWN miRNAs^66^, allowing for the maximum of 2 mismatches. Novel miRNAs were predicted using a minimum hairpin length of 50.

Differential expression analysis was performed for *P. pinaster* miRNAs using DESeq2^67^ with a 0.05 false discovery rate (FDR) threshold. To identify miRNAs responsive to PWN inoculation, inoculated plants were compared to control plants, while to identify miRNAs possibly involved in resistance, susceptible samples were compared to resistant ones. CPMs (Counts Per Million) were calculated for each sample by normalizing against the total number of reads in each library and multiplying by a factor of 10^6^. These CPMs were used to create expression heatmaps in R v4.1.0 (https://www.R-project.org/).

### Target prediction and enrichment analysis

Prediction of miRNA targets in *P. pinaster* was performed using the online tool psRNTarget^68^ with default parameters (except for *HSP size* = 18), and *P. pinaster* transcriptome, containing only transcripts with predicted coding sequences^13^. As mRNA transcription data was available for the same samples as the ones analysed in this paper^13^, it was possible to correlate the expression of the sRNAs and their predicted target genes. Pearson correlations were calculated using R and only pairs of sRNA-targets with expressions negatively correlated (R ≤ − 0.65) were retained. Targets were predicted for *P. pinaster* miRNAs and tasiRNAs, as well as PWN miRNAs.

To validate the targeting of *P. pinaster* transcripts by PWN miRNAs, degradome sequencing data available in European Nucleotide Archive (ENA) database (PRJEB48279) were used. These data consist of two libraries containing a pool of RNA extracted from stem samples of four resistant and four susceptible *P. pinaster* samples at 72 hpi. Although these samples belong to a different family than the one used in the present study, family 465^9^, the inoculum used and the collection timepoint were the same and therefore, variation in PWN miRNA expression is expected to be low. Degradome sequencing data, PWN detected miRNAs, and *P. pinaster* transcriptome^13^ were used as input for CleaveLand4 v4.5^69^ to detect cleaved sRNA targets.

Target genes were predicted in PWN using miRanda v3.3a^70^, with a minimum score of 120 and maximum energy of − 20. For this analysis, only 3′ UTR sequences were used (up to 800 bp upstream from the predicted coding sequences), as in animals miRNAs target primarily these regions, and not the entire gene^71^. Targets were predicted for *P. pinaster* DE miRNAs.

PWN genome was functionally annotated by aligning sequences with NCBI RefSeq Invertebrate database (accessed May 2021) using BLASTx in DIAMOND v2.0.9^72^. InterProScan was used to attribute gene ontology (GO) terms and Kyoto Encyclopedia of Genes and Genomes (KEGG) pathways^26,27^. KEGG annotation was further improved by using KEGG Automatic Annotation Server (KAAS)^73^. GO and KEGG annotations for *P. pinaster* transcriptome were obtained from Modesto et al*.*^13^.

For the predicted target genes, for both *P. pinaster* and PWN, gene set enrichment analysis was performed with BiNGO plugin^74^ for Cytoscape^75^, using the hypergeometric statistical test and Benjamini and Hochberg FDR for multi testing correction (*p*-value ≤ 0.05). Gene ontology redundancy was reduced using the online tool Revigo^76^ with a trim threshold of 50%. Pathway enrichment analysis and Pfam enrichment analysis were made with BiNGO using the same parameters as described above.

### RT-qPCR

Five *P. pinaster* miRNAs DE between inoculated and control samples were selected for expression profile validation, together with five predicted target genes negatively correlated with these miRNAs, according to previously published RNA-seq data^13^. cDNA of three resistant, three susceptible, and three control samples was synthesized using Mir-X miRNA First-Strand Synthesis Kit (Takara Bio, USA). Forward primers were manually designed to match the entire sequence of the miRNA to be amplified (Supplementary Table S16), while the reverse primer used was the universal mRQ 3’ primer supplied with the kit. For the target genes, primers were designed with PerlPrimer v1.1.21^77^ (Supplementary Table S16). RT-qPCR was run in a LightCycler 480 Instrument II (Roche, Switzerland) using SYBR Green I Master (Roche) and the following conditions: 5 min at 95 °C, 40 cycles of 95 °C for 10 s, 61–66 °C for 15 s (Supplementary Table S16), and 72 °C for 12 s. Primer specificity was monitored by analysing the melting curves. Three technical replicates were performed for each biological replicate. Expression profiles were normalized using *5S rRNA* as a reference for miRNAs, while *actin*, *40S rRNA*^78^ and *histone H3*^79^ were used for the target genes. Relative expression levels were calculated with the Pfaffl method^80^. Pearson’s correlation coefficient was calculated between RNA-seq and RT-qPCR expression levels [log2(fold change)] in R, for both miRNAs and target genes. Correlation analysis was also performed between the RT-qPCR expression levels [log2(fold change)] of miRNAs and respective predicted target genes. Significance of these results was obtained through a correlation test (*t*-test) in R.

## Supplementary Information

Supplementary Tables.

Supplementary Figures.

## Data Availability

The sequence data for this study has been submitted to the European Nucleotide Archive (ENA) under Accession Number PRJEB48441 (https://www.ebi.ac.uk/ena/browser/view/PRJEB48441).
