# Supplementary material for: MicroRNA-mediated post-transcriptional regulation of Pinus pinaster response and resistance to pinewood nematode
Source: Sci Rep. 2022 Mar 25;12:5160. doi: 10.1038/s41598-022-09163-3 (PMC8956650; doi:10.1038/s41598-022-09163-3)
Supplement: Supplementary file 2 — Supplementary Figures. [file 41598_2022_9163_MOESM2_ESM.pdf]

MicroRNA-mediated post-transcriptional regulation of *Pinus pinaster* response and resistance to pinewood nematode

Inês Modesto, Vera Inácio, Yves Van de Peer, Célia M. Miguel

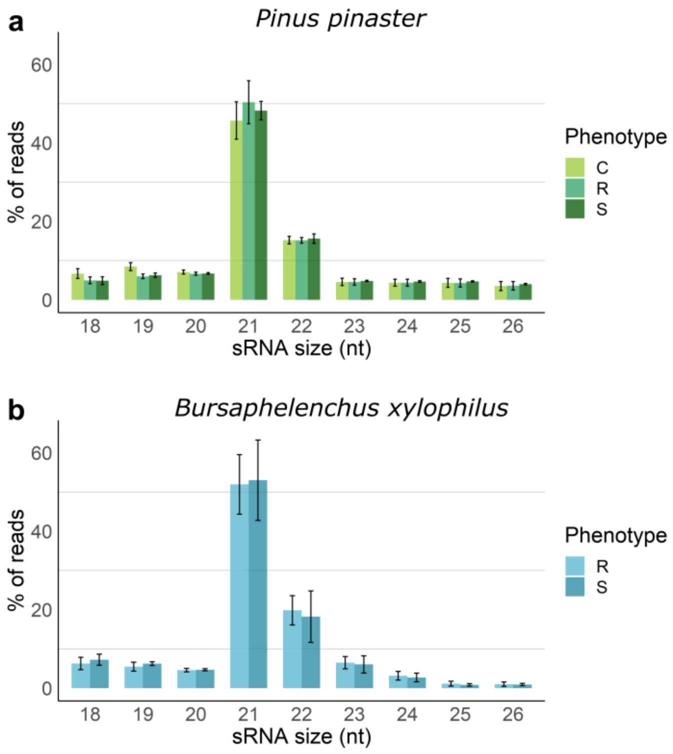

Supplementary Figure 1. Sizes of *P. pinaster* (a) and PWN (*B. xylophilus*) (b) sRNAs after filtering the reads, in resistant (R), susceptible (S), and control (C) samples. The y-axis represents the percentage of reads in each size ~~(in nucleotides, nt) category~~. Plots were generated with R 4.1.0 (https://cran.r-project.org/) ggplot2 package (https://ggplot2.tidyverse.org/). Inkscape 1.1 (https://inkscape.org/) was used to assemble the final figure.

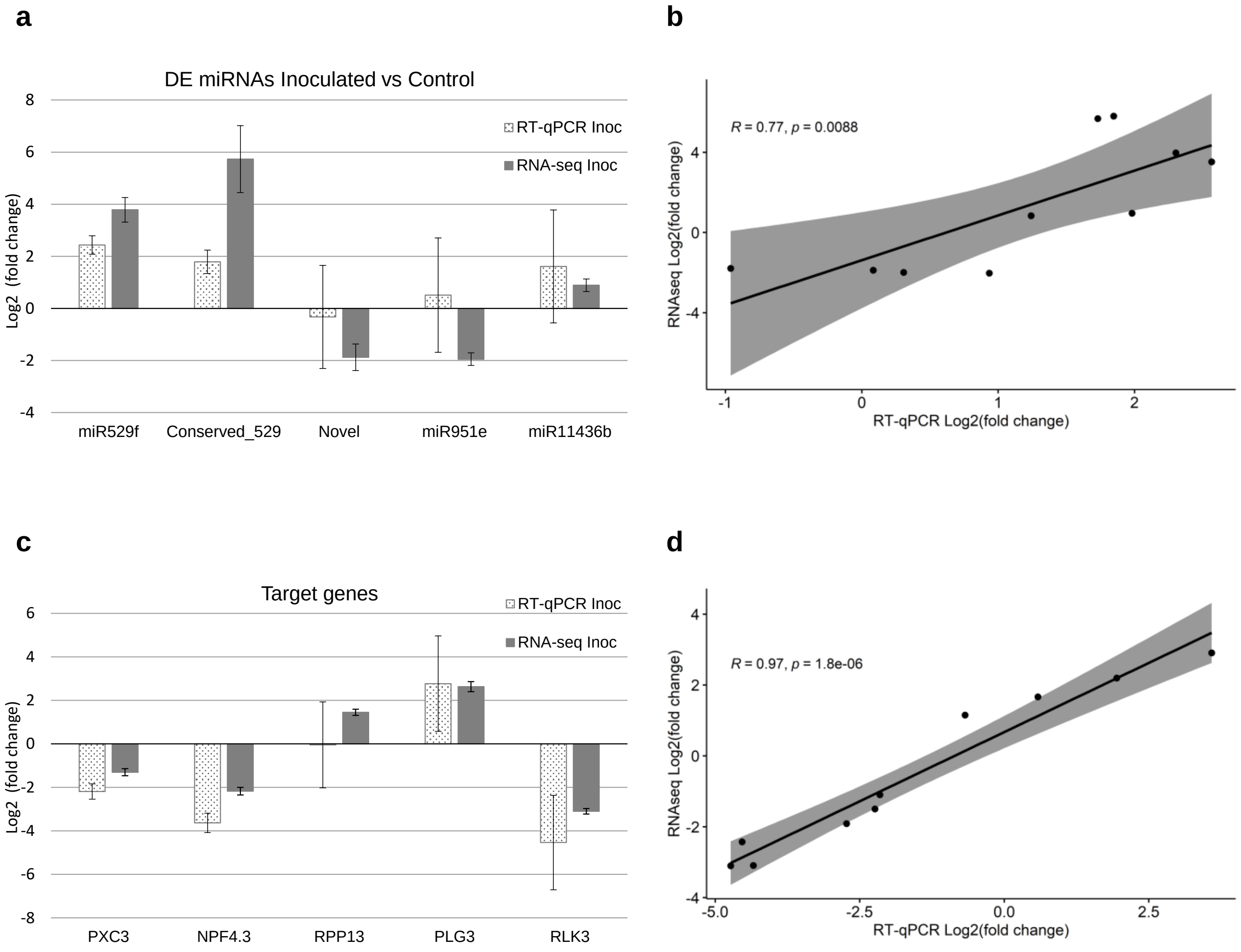

Supplementary Figure 2. RT-qPCR analysis of 5 DE miRNAs and 5 predicted target genes. (a)(c) Bars represent differential expression levels, in log2(fold change), of inoculated plants in comparison with controls. Results from both the RNA-seq analysis (filled color) and the RT-qPCR analysis (dots) are displayed. Error bars represent the standard error of the biological replicates used for RNA-seq (4–5) and RT-qPCR (3). (b)(d) Pearson's correlation analysis of expression levels [log2 (fold change)] between RNA-Seq and RT-qPCR. Plots were generated with Image generated with Microsoft Office Excel and with R 4.1.0 (https://cran.r-project.org/) ggplot2 package (https://ggplot2.tidyverse.org/). Inkscape 1.1 (https://inkscape.org/) was used to assemble the final figure.
